# A new risk and issue management system to improve productivity, quality, and compliance in clinical trials

**DOI:** 10.1093/jamiaopen/ooz006

**Published:** 2019-03-19

**Authors:** Joseph Ciervo, Shih Chuan Shen, Kristin Stallcup, Abraham Thomas, Michael A Farnum, Victor S Lobanov, Dimitris K Agrafiotis

**Affiliations:** Covance, the Drug Development Division of LabCorp, Princeton, New Jersey, USA

**Keywords:** risk management, issue management, risk-based monitoring, Xcellerate, clinical informatics, clinical trial

## Abstract

**Objective:**

We present a new system to track, manage, and report on all risks and issues encountered during a clinical trial.

**Materials and Methods:**

Our solution utilizes JIRA, a popular issue and project tracking tool for software development, augmented by third-party and custom-built plugins to provide the additional functionality missing from the core product.

**Results:**

The new system integrates all issue types under a single tracking tool and offers a range of capabilities, including configurable issue management workflows, seamless integration with other clinical systems, extensive history, reporting, and trending, and an intuitive web interface.

**Discussion and Conclusion:**

By preserving the linkage between risks, issues, actions, decisions, and outcomes, the system allows study teams to assess the impact and effectiveness of their risk management strategies and present a coherent account of how the trial was conducted. Since the tool was put in production, we have observed an increase in the number of reported issues and a decrease in the median issue resolution time which, along with the positive user feedback, point to marked improvements in quality, transparency, productivity, and teamwork.

## BACKGROUND AND SIGNIFICANCE

The growing size and complexity of clinical trials and the increased regulatory scrutiny placed upon study teams have accentuated an important gap in study management, namely, the ability to track, manage, document, and report on all risks and issues encountered during the conduct of a trial in a holistic and auditable manner. Typically, risks manifest themselves as issues, issues trigger actions, actions lead to decisions, and decisions produce outcomes. Maintaining that lineage allows study teams to assess the impact and effectiveness of their risk management strategies and ultimately tell the story of how the trial was conducted.

Historically, that story has been hard to weave due to the absence of a holistic risk management system that covered all relevant functional areas and met the needs of the entire study team. Existing tools track only a portion of potential risks, are aimed at only a fraction of the study team, are applicable to only a subset of activities, or lack essential collaboration capabilities. Auditors have reported several recurrent problems, including teams not being able to find the rationale behind important decisions, escalations not being done in a timely manner, and communications between the sponsor and their partner(s) being inadequately documented. In one example (the details of which cannot be disclosed due to our confidentiality agreement with the sponsor), a study manager lamented that a missed escalation of an important issue resulted in 4 million dollars of extra spend due to rework, when a simple on-time escalation could have prevented it. While the impact of these problems is not always as quantifiable, everyone is familiar with searching emails for an important piece of information or wishing there were a better way to learn from past experience.

Another reason that necessitates a more cohesive approach is the advent of risk-based monitoring (RBM).^1–5^ Most clinical study teams tend to work in silos, leading to miscommunication and duplication of effort. Before RBM, the person who identified an issue was generally responsible for addressing it, leading to poor oversight and increased risk. RBM, through its emphasis on risk management, provides the impetus to manage risks throughout their lifecycle, which includes issue management. Operationally, RBM introduces an additional layer of central monitors who perform a holistic assessment of risk to identify issues across the entire study and assign them to other team members for follow-up and resolution. This process requires comprehensive access to issues and their status, and efficient workflows to resolve them.

These considerations have led regulatory agencies and industry consortia to emphasize the need for a more rigorous and centralized approach to risk and issue management (RIM), the basic tenets of which are outlined in the E6 (R2) addendum to the International Council for Harmonization Good Clinical Practice guidelines (ICH GCP E6 [R2])^4^ and the TransCelerate framework on clinical quality management.^6^ These updated guidelines recommend that study teams focus their efforts on “issues that matter,” that is, issues that “materially impact patient safety, rights, and well-being; data integrity and/or scientific rigor; compliance with regulatory requirements; or trust in the clinical research enterprise.” Meeting these guidelines requires new systems and processes that cover the entire lifecycle and scope of risk management, including identification, mitigation, reporting, and analysis. Some leading software vendors have begun to address this need in their latest offerings, leveraging in most cases their existing clinical trial management systems (CTMS).^7–9^

In designing such a system, a number of considerations need to be taken into account: study risks are identified proactively before they occur; when realized, risks become issues; risks and issues are mitigated through actions and lead to decisions; protocol deviations are a special form of issues; and risks and learnings from previous trials can be applied to new studies. As stated above, maintaining the linkages between risks, actions, protocol deviations, issues, and decisions (which we collectively call RAPIDs) gives study teams the ability to reconstruct events during an audit and identify repeating issues that may suggest a systemic problem or trend. Detecting such trends allows teams to address the root cause of a problem before it becomes widespread and avoid the same mistake in future trials. The requirements for communication and management of risks, issues, and protocol deviations are outlined in the study plans, which also include workflows for reviewing these items and rules for escalation. To demonstrate compliance, the system must be comprehensive; to drive compliance, it must also be intuitive.

Recently, we introduced a comprehensive application suite, known as Xcellerate, that uses advanced data integration, analytics, and visualization capabilities to improve patient safety, data quality, and protocol compliance throughout the clinical development process and enable greater transparency and oversight of study conduct and performance.^10^^,^^11^ The solution consists of a number of end-user applications connected to a clinical data repository that supports near-real-time acquisition, mapping, and integration of clinical trial data from any germane source.^12^^,^^13^ One of these applications is the RIM system described herein. The Xcellerate RIM unifies all issue types under a single tracking system and offers a broad range of capabilities, including customizable and extensible issue types, configurable issue management workflows for entry, review, assignment, delegation, escalation, and closure, support for annotations, comments, and attachments, convenient issue linking and cross-referencing, flexible handling of alerts and notifications, extensive search capabilities, single sign-on and user management with granular roles and permissions, extensive history, metrics reporting, and trending, seamless integration with other clinical systems, full audit trail, and an intuitive web interface to enhance user adoption and productivity.

## METHODS

These requirements bear many similarities to the issue and task tracking software that has been used in the software industry for more than 20 years to manage complex software development projects. This software was created and enhanced by developers to enable collaboration among multiple, often distributed teams, and has become robust, flexible, and ubiquitous. Our team has used a popular issue tracking tool, Atlassian JIRA,^14^ to manage our daily agile development work for more than 10 years^15–18^ and has developed a lot of experience and familiarity with it, making it a natural starting point in our build-versus-buy decision.

JIRA is an easy-to-use and highly adaptable tool due to its extensive configuration and extensibility capabilities. Besides its use in software development projects, several business teams have adopted the tool for their own, distinct use cases.^19–21^ Furthermore, JIRA has a large and thriving user community and ecosystem, which instills confidence about its long-term viability and support.

JIRA’s architecture is standards-based, modular, and open. The application is written in Java and runs in a standard Tomcat Java Servlet Container. The system components include a relational data store, a data access layer, workflow services, authentication services, authorization services, scheduling services, index and search services, user and administrator interfaces, and an extensible plugin framework. Additionally, the software offers extensive REST and Java Application Programming Interfaces (APIs) that provide access to virtually all its capabilities to facilitate integration and extension of the product. The plugin framework and open APIs were a key factor in our decision to adopt JIRA as the foundation for RIM. Many of JIRA’s core features are themselves implemented as plugins using these same APIs, and many plugins are configurable in their own right, offering a wealth of options to extend the core product.

In adapting JIRA to meet our requirements, our preference was to use the out-of-the-box features with default settings whenever possible, followed by enhancement through JIRA’s built-in configuration capabilities, third-party plugins, and custom plugins, in that order. Specifically:

- We used JIRA’s out-of-the-box capabilities to implement a project-based, configurable web application framework with project-level navigation and security; REST APIs for integration with other clinical systems; full text search within and across projects; field level filtering and querying within and across projects; Excel, Word, and PDF export of issues; audit trail and history tracking; user management and role assignment; email notifications for reporters, assignees, and other watchers; issue-level commenting; and basic reporting.
- We used JIRA’s configuration capabilities to implement RAPID-specific forms; RAPID-specific workflows; workflow-state-specific forms; granular permissions for clinical study teams; and RAPID linking options.
- We purchased third-party plugins to implement security at the RAPID field level; custom form fields that can display study-specific content from existing web services while maintaining study level security; Security Assertion Markup Language single sign-on and identity federation support; and configuration and deployment on multiple environments.
- We developed custom Java plugins to implement manual and automatic rule-based escalations; site bulk action creation; RAPID type conversion; dependent form fields; display of domain-specific instead of generic project management nomenclature in the user interface; use of Xcellerate branding; use of a Java Messaging Service (JMS) module to support CTMS integration using the TIBCO Enterprise Service Bus (ESB); and integration with legacy extract-transform-load (ETL) tools.
- Finally, we used a combination of configurations and custom plugins to disable and hide unused features of the base JIRA product.

While RIM can be used as a standalone application, its benefits are amplified when used in conjunction with the broader Xcellerate suite.^10^ We have currently implemented the following integrations: (1) 2-way real-time integration with Oracle Siebel CTMS through the TIBCO ESB using a custom JMS plugin; (2) 2-way real-time integration with the Xcellerate applications using the .Net Software Development Kit and JIRA’s REST APIs; and (3) ETL integration with the Xcellerate Operational Data Warehouse^12^ using custom transformation middleware.

## RESULTS

**Figure 1.**
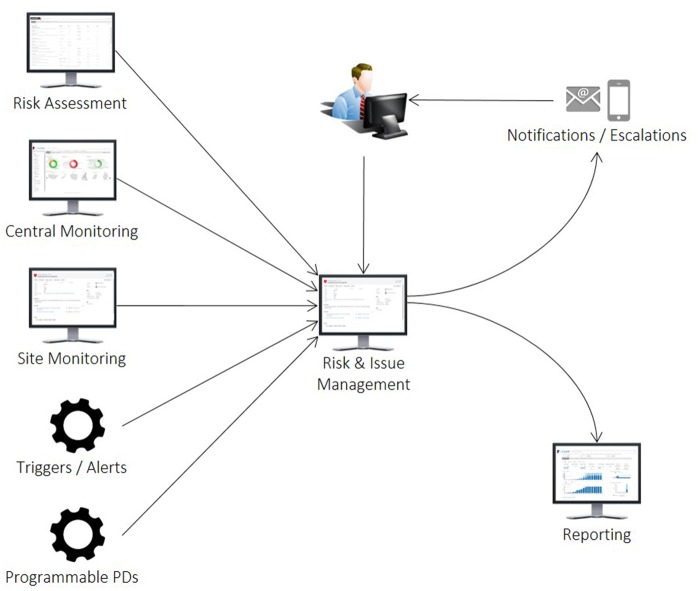
Integration of the Xcellerate risk and issue management system into the clinical trial monitoring workflow. RAPIDs can be created manually through risk assessment, central monitoring, and site monitoring activities, or automatically through preprogrammed triggers. RAPID: risks, actions, protocol deviations, issues, and decisions.

**Figure 2.**
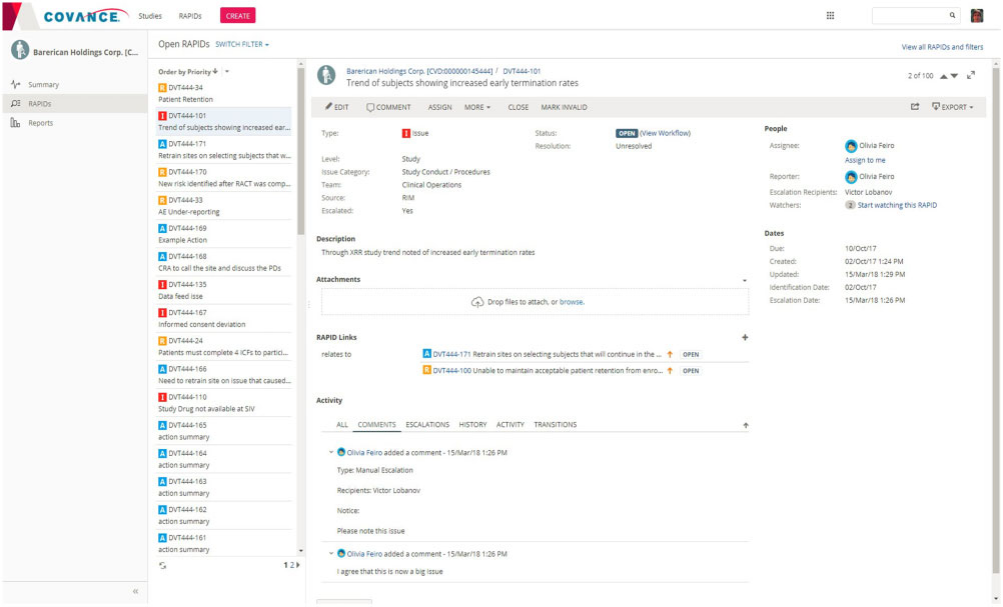
Representative screenshots of the Xcellerate risk and issue management application.

The RAPID flow is illustrated in Figure 1 and representative screenshots of the Xcellerate RIM application are shown in Figure 2. The tool simplifies clinical trial management by providing a single place for managing risks and issues of any type, across all functional areas, systems, and processes, and throughout the life of the study. RIM breaks down functional silos by introducing a holistic and collaborative approach to issue management and eliminates the need for multiple tracking spreadsheets, meeting minutes, and email logs that have been a constant source of frustration, inefficiency, error, and noncompliance.

**Figure 3.**
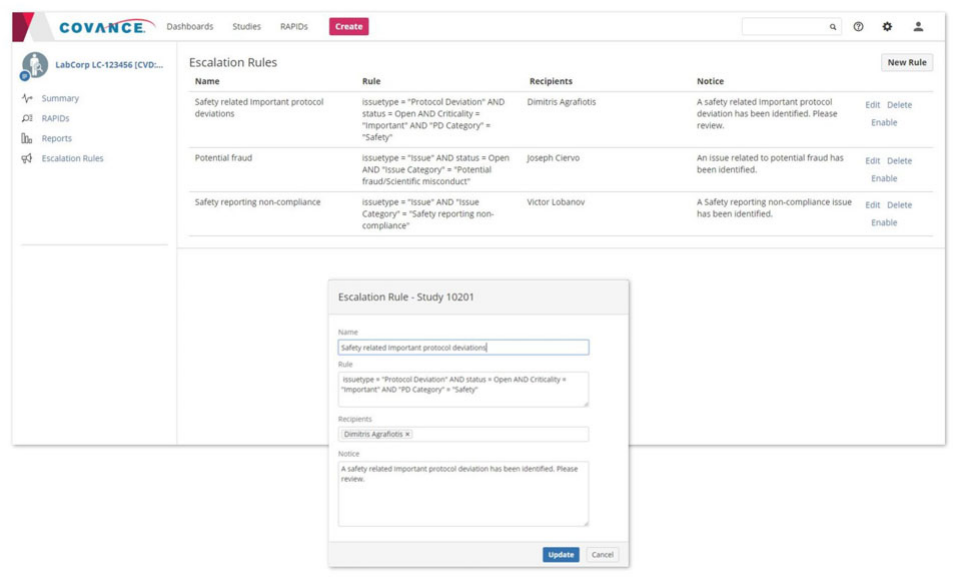
Rule-based escalation of important issues and protocol deviations.

The use of incremental comments enables discussions among study team members to be kept organized, documented, and readily available. The most important discussions relate to the escalation of issues to the clinical team leader(s) and the sponsor. Before RIM, ensuring that these escalations happened on time was extremely difficult. Clinical research associates would often forget whom to escalate an issue to and complain that their escalations went into a “black hole” and never received a response. In addition to manual escalations which can be raised at any time, RIM allows study teams to set up escalation rules at the start of the trial so that important issues and protocol deviations are automatically escalated to a designated individual upon creation, and delivered through alerts and email notifications (Figure 3). The assignee can either manage the item within the system or respond directly through email. In both cases, the response is captured within RIM and is visible on the discussion thread. This built-in escalation workflow ensures compliance to response timelines and rigorous record-keeping for audit purposes.

RIM provides a full audit trail, including the name, date, and time stamp for any creation, update, review, escalation, or other activity in the system. This greatly enhances compliance to ICH GCP E6 (R2) guidelines and further promotes collaboration, as study team members know that their contributions are visible and permanently logged. Auditors often ask for proof that reviews and escalations were done on time. RIM allows that information to be exported quickly and easily to demonstrate compliance during an audit.

RIM can integrate with any conceivable issue source and provides customizable workflows for different issue types. Integration with the Xcellerate application suite^10^ (Risk Review, Risk Assessment and Categorization Tool, Medical Review, Statistical Review, Data Review, CRA Dashboard), CTMS, and other systems minimizes manual data entry and ensures a closed loop from identification to closure. This integration is not a prerequisite, since RIM can work as a standalone application. Our licensing structure, which includes Jira licensing fees, is designed to fit the needs and budgets of any company, regardless of size.

Importantly, RIM maintains the integrity and lineage of events and decisions, which gives study teams the opportunity to understand the downstream impact of their mitigation strategies, learn from past experiences, and apply those learnings to new clinical trials. It also enables the application of machine learning techniques to develop predictive risk models that can be continuously refined through an ever-growing volume of consistently captured data.

## DISCUSSION

The system was released to production in July 2017 and following a 12-month pilot with 15 early adopter studies, was mandated for all new fully outsourced trials (for functionally outsourced trials, we typically leverage the sponsor’s systems and processes). As of this writing, RIM is being utilized in 36 studies, 22 of which have begun reporting RAPIDs. In addition to the very positive feedback received from our study teams, we have noticed a marked increase in the number of risks, issues, and escalations reported through RIM and, more importantly, a meaningful and statistically significant reduction (30%) in the amount of time required to resolve issues (see Table 1). We believe this is due to the increased transparency, awareness, and ability to escalate issues according to study plans. For some team members, knowing that the rest of the study team and the sponsor have access and visibility into “their issues” can at first be a little unsettling. However, user feedback indicates that these concerns are offset by the additional convenience afforded by the system, and completely dissipate as the benefits in quality, productivity, and compliance become more clear.

**Table 1.** Comparison of issue resolution time (from creation to closure) for studies run with (RIM = Y) and without (RIM = N) the use of the Xcellerate RIM system

RIM	Issue count	Study count	Med days to resolve	Avg days to resolve	StdDev days to resolve
N	716 164	956	60	100	129.7
Y	1077	14	42	87	116.0

*Note:* This includes all historical studies at Covance captured in the Xcellerate Operational Data Warehouse. The 2-tailed *t*-test of the 2 population means has a *P*-value of <0.0001, suggesting a meaningful and statistically significant reduction in the mean issue resolution time for studies that used the RIM system.

RIM: risk and issue management.

## CONCLUSION

We have presented the technical underpinnings of a new RIM system, along with supporting evidence which suggests that the new system can improve user adoption, productivity, quality, transparency, and compliance. The system meets regulators’ expectations for sponsors and their providers to clearly articulate their patient safety and data quality management strategies with more definitive, rigorous, and documented procedures that codify risk management, and demonstrate that these plans have been executed correctly. Planned enhancements include a mobile version of the application, ability to make offline updates, and incorporation of workflows specific for corrective and preventative action management.

## AUTHOR CONTRIBUTORS

JC and VSL conceptualized the invention, led the design, development and implementation of the software, and contributed to the manuscript. SCS and MAF contributed to the design, development, and implementation of the software. KS provided user requirements and contributed to the manuscript. AT contributed to the design, development, testing, deployment, and support of the software. DKA was accountable for the work, contributed to the manuscript, and approved the final manuscript as submitted.
